# Shared transcriptional regulators and network rewiring identify therapeutic targets linking type 2 diabetes mellitus and hypertension

**DOI:** 10.3389/fmolb.2025.1621413

**Published:** 2025-08-20

**Authors:** Claudia Desireé Norzagaray-Valenzuela, Marco Antonio Valdez-Flores, Josue Camberos-Barraza, Alberto Kousuke De la Herrán-Arita, Juan Fidel Osuna-Ramos, Javier Magaña-Gómez, Carla Angulo-Rojo, Alma Marlene Guadrón-Llanos, Katia Aviña-Padilla, Loranda Calderón-Zamora

**Affiliations:** ^1^ Faculty of Biology, Autonomous University of Sinaloa, Culiacan, Mexico; ^2^ Graduate Program in Biological Sciences, Faculty of Biology, Autonomous University of Sinaloa, Culiacan, Mexico; ^3^ Faculty of Medicine, Autonomous University of Sinaloa, Culiacan, Mexico; ^4^ Graduate Program in Molecular Biomedicine, Faculty of Medicine, Autonomous University of Sinaloa, Culiacan, Mexico; ^5^ Faculty of Nutrition, Autonomous University of Sinaloa, Culiacan, Mexico; ^6^ Department of Genetic Engineering, Center for Research and Advanced Studies of the National Polytechnic Institute (CINVESTAV-IPN), Irapuato, Mexico

**Keywords:** type 2 diabetes mellitus, hypertension, transcriptomics, Interactomics, coexpression networks, inflammatory pathways, vascular remodeling

## Abstract

**Background:**

Type 2 diabetes mellitus (T2DM) and Hypertension (HTN) frequently coexist and synergistically exacerbate vascular and immune dysfunction. Despite their clinical interrelation, these diseases have traditionally been studied in isolation, and the molecular mechanisms underlying their comorbidity remain poorly understood. This study aimed to uncover shared transcriptional programs and disease-specific regulatory networks contributing to cardiometabolic dysfunction.

**Methods:**

We systematically selected transcriptomic datasets and employed an integrative systems biology approach that combined differential gene expression analysis, co-expression network construction, protein-protein interaction mapping, transcription factor activity inference, and network rewiring analysis. Functional enrichment analyses were conducted to elucidate biological processes associated with disease-specific modules.

**Results:**

We identified distinct regulatory modules: ME3 in T2DM, enriched in metabolic stress response, intracellular trafficking, and inflammation, and ME7 in HTN, enriched in immune response and vascular remodeling. Protein interaction networks revealed key hub genes such as *GNB1*, *JAK1*, and *RPS3* as T2DM-specific hubs, while MAPK1, *BUB1B*, and RPS6 were central in HTN. Network rewiring analysis showed condition-specific changes in gene connectivity, particularly in *ST18* and *SLBP* gaining prominence in T2DM, and *SLC16A7* and *SPX* showing decreased connectivity in HTN. Notably, transcription factor activity analysis revealed shared regulators *HNF4A* and *STAT2* implicated in inflammation, oxidative stress, and vascular remodeling, highlighting a transcriptional convergence between the two conditions.

**Conclusion:**

This study provides novel insights into the molecular crosstalk between T2DM and HTN by identifying conserved transcriptional regulators and rewired gene networks. Our findings support the existence of a shared regulatory architecture underlying cardiometabolic comorbidity and suggest promising diagnostic and therapeutic targets for precision medicine.

## 1 Introduction

Type 2 diabetes mellitus and hypertension are complex, multifactorial conditions that frequently co-occur and synergistically exacerbate vascular, renal, and metabolic dysfunction, leading to accelerated cardiovascular disease progression (Grundy, 2006; De Boer et al., 2017; Petrie et al., 2018). Although traditionally investigated as distinct clinical entities, emerging evidence points to a significant overlap in their molecular pathogenesis, including shared transcriptional programs and regulatory circuits (Hasin et al., 2017; Jeyananthan et al., 2025). This convergence suggests that both diseases may be driven, at least in part, by common gene expression networks that modulate inflammation, endothelial remodeling, and metabolic dysregulation (Hotamisligil, 2006; Barabási et al., 2011; Hasin et al., 2017). Despite this, the precise molecular mechanisms orchestrating their interaction remain insufficiently characterized. Elucidating these convergent and disease-specific transcriptional signatures is essential for uncovering regulatory hubs with diagnostic and therapeutic relevance and may provide a framework for the development of precision medicine strategies targeting cardiometabolic comorbidity.

T2DM, which accounts for over 90% of diabetes cases, is characterized by progressive β-cell dysfunction, insulin resistance, and systemic metabolic disturbances (ElSayed et al., 2023). The global burden of T2DM is expected to surpass 783 million cases by 2045, resulting in more than 6.7 million diabetes-related deaths annually (Magliano and Boyko, 2021). HTN, now defined by the ACC/AHA 2017 guidelines as systolic blood pressure ≥130 mmHg or diastolic pressure ≥80 mmHg, arises from vascular remodeling, neurohormonal imbalance, and renal dysfunction (Greenland and Peterson, 2017; Oparil et al., 2018). Affecting more than one billion people worldwide, HTN contributes to approximately 10.8 million deaths each year (Jeemon et al., 2021).

Despite differences in clinical presentation, T2DM and HTN share fundamental pathophysiological mechanisms, notably insulin resistance and overactivation of the renin-angiotensin-aldosterone system (RAAS). Insulin resistance, a hallmark of T2DM, impairs endothelial function, promotes sodium retention, and enhances sympathetic nervous system activity, collectively contributing to elevated blood pressure and vascular dysfunction (Zhou et al., 2010). In parallel, RAAS hyperactivity, present in both conditions, exacerbates vascular remodeling, oxidative stress, and chronic low-grade inflammation (Jandeleit-Dahm and Cooper, 2006). These interconnected mechanisms reinforce one another, promoting cardiometabolic deterioration. Building upon these shared physiological pathways, recent advances in genomics have identified disease-relevant transcriptional regulators that may underline the complex interplay between metabolic and vascular dysfunction.

Genetic studies have identified key transcriptional regulators implicated in the pathogenesis of both T2DM and HTN, offering insights into their shared and disease-specific molecular networks. In T2DM, genes involved in metabolic control, such as *TCF7L2* and *PPARG*, play central roles in insulin secretion and glucose homeostasis (Sarhangi et al., 2020; Del Bosque-Plata et al., 2021). Additionally, variants in *KCNJ11* and *SLC30A8*, which influence pancreatic β-cell excitability and insulin storage, have increased disease susceptibility (Proks et al., 2004; Sladek et al., 2007; Lemaire et al., 2009). These findings underscore the contribution of transcriptional dysregulation to metabolic imbalance and β-cell failure (Voight et al., 2010).

Conversely, HTN is primarily driven by genes regulating vascular tone, endothelial integrity, and blood pressure homeostasis. Components of the RAAS pathway, such as *AGT* and *ACE*, modulate blood pressure through angiotensin-mediated vasoconstriction and fluid balance (Purkait et al., 2017; Zambrano et al., 2023). Moreover, ion channel–encoding genes such as *SCN7A*, *KCNMA1*, and *CACNA1C* have been linked to abnormalities in vascular contractility and autonomic regulation (Saleh et al., 2005; Wu and Marx, 2010; Pereira da Silva et al., 2022). Additional regulators, such as *NOS3* and *CYP11B2*, further highlight the importance of nitric oxide bioavailability and aldosterone synthesis in hypertensive pathophysiology (Davies et al., 1999; Oliveira-Paula et al., 2016).

Although numerous molecular determinants of T2DM and HTN have been individually characterized, the extent to which these diseases share interconnected regulatory architectures remains poorly understood. Previous systems biology studies in other cardiometabolic disorders have demonstrated the value of transcriptomic and network-based approaches in uncovering hidden molecular interactions and shared regulatory pathways (Barabási et al., 2011; Hasin et al., 2017). However, integrative analyses that simultaneously address the transcriptional landscapes of T2DM and HTN remain limited.

Multi-scale systems biology approaches have recently emerged as essential frameworks for dissecting the complexity of chronic diseases by integrating multiple layers of molecular information. In particular, transcriptomic analyses provide a high-resolution view of gene expression changes and their functional implications, enabling the identification of disease-relevant pathways and regulatory programs (Khan and Kim, 2024). Complementarily, interatomic analyses, such as protein-protein interaction (PPI) networks, reveal the structural and functional organization of cellular systems, exposing central regulatory hubs and potential therapeutic targets (Sun and Hu, 2016). By leveraging these complementary strategies, our study aims to dissect the transcriptional convergence between T2DM and HTN and to uncover molecular signatures that may serve as therapeutic targets. Integrating transcriptomic and interactomic approaches enhances our understanding of how metabolic and vascular reprogramming contributes to disease pathology and lays the groundwork for precision medicine strategies targeting shared and disease-specific regulatory mechanisms in complex disorders.

## 2 Materials and methods

**FIGURE 1:**
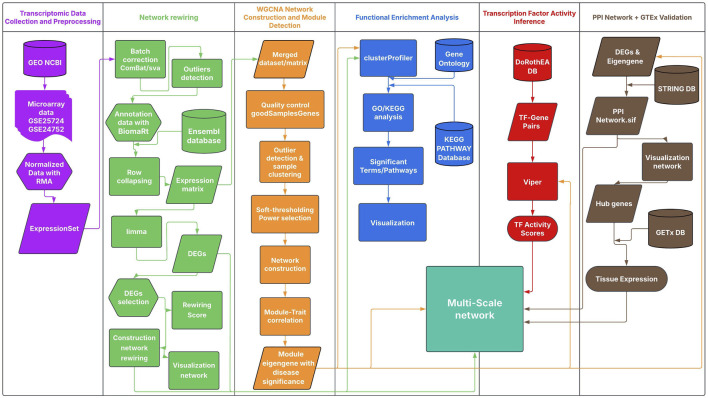
Multi-Scale Network Analysis Workflow for T2DM and HTN. The workflow integrates distinct analytical stages; each color-coded as in the Figure: Transcriptomic data preprocessing (purple), including normalization of GEO microarray data; Network rewiring analysis (green), with differential expression (limma) and rewiring score calculation; WGCNA-based module detection (orange), identifying trait-associated gene modules; Functional enrichment analysis (blue) using GO and KEGG with clusterProfiler; Transcription factor activity inference (red) via DoRothEA and VIPER; and PPI network analysis (brown) using STRING. Potential genes were validated using GTEx DB.

This study employed a multi-scale network approach to analyze transcriptomic data and identify predictive hub genes associated with complications in T2DM and HTN (Figure 1). The analysis integrated differential gene expression profiling and weighted gene co-expression network analysis (WGCNA) to identify disease-associated modules. Functional enrichment analyses were conducted on differentially expressed genes and co-expression modules to elucidate relevant biological processes. Additionally, to capture context-specific rewiring of gene interactions, we quantified connectivity gains and losses between conditions and retained significant rewiring events. Transcription factor (TF) activity inference was used to uncover regulatory mechanisms, and protein-protein interaction (PPI) networks were constructed to identify central molecular hubs. Community detection within the PPI networks enabled a second layer of functional enrichment, focusing on the most interconnected gene clusters. *In silico* validation of candidate genes was performed to assess their tissue-specific expression profiles. Altogether, this multi-scale network approach provided a comprehensive view of the shared regulatory mechanisms underlying T2DM and HTN. All scripts used in this analysis are available in this GitHub repository.

### 2.1 Systematic identification and selection of transcriptomic DataSets for the study of T2DM and HTN

A systematic search was conducted in the National Center for Biotechnology Information (NCBI) Gene Expression Omnibus (GEO) public repository to identify microarray-based transcriptomic datasets suitable for comparative gene expression analysis in T2DM and HTN. The search employed the terms “*type 2 diabetes mellitus”* and *“essential hypertension,”* with filters applied for *Homo sapiens*, expression profiling by array, CEL file availability, case-control design, and relevant tissues (pancreatic islets for T2DM and kidney tissue or peripheral blood for HTN). Three of the eleven initially identified datasets for T2DM met all selection criteria: GSE25724, GSE20966, and GSE38642. However, GSE38642 was excluded due to its use of pancreatic islets obtained from *postmortem* donors, which may compromise RNA quality and biological interpretability. For HTN, three datasets (GSE24752, GSE28345 and GSE28360) fulfilled the inclusion criteria. Based on these selections, three integrative expression matrices were constructed: one for T2DM, one for HTN, and a final combined matrix integrating selected datasets from both conditions to allow cross-disease comparative analyses.

### 2.2 Transcriptomic data collection, preprocessing, and analysis strategies

Microarray datasets GSE25724 (T2DM; 13 samples: six cases and seven controls), GSE20966 (T2DM; 20 samples: ten diabetic and ten controls), GSE24752 (HTN; six samples: three normotensive and three hypertensive), GSE28345 (HTN; 8 samples: five hypertensive and three controls), and GSE28360 (HTN; 14 samples: nine hypertensive and five controls) were retrieved from the GEO database (https://www.ncbi.nlm.nih.gov/geo/, accessed on 7 January 2025). Raw CEL files and associated metadata were downloaded, extracted, and processed using R version 4.3.3 within the RStudio environment. Expression values were normalized using the Robust Multi-array Average (RMA) method. Quality control procedures, including hierarchical clustering and multidimensional scaling (MDS), were applied to detect outlier samples and assess potential batch effects. Genes with low variance were filtered out to retain biologically informative features. Two complementary analytical strategies were applied: 1) *Integrated analysis:* the normalized expression matrices from all selected datasets were merged based on common genes across platforms. Prior to integration, data were normalized using quantile normalization and corrected for batch effects to ensure comparability across samples. Sample and gene quality were assessed using the goodSamplesGenes function from the WGCNA package, retaining genes above the 70th percentile of variance. A combined co-expression network was constructed to detect modules associated with clinical traits common to both conditions, and 2) *Disease-specific analysis*: each dataset was processed independently to capture transcriptional signatures unique to each condition. For both T2DM and HTN, the optimal soft-thresholding power (β) was determined using the pickSoftThreshold function from WGCNA. The β values were chosen based on their corresponding scale-free topology model fit (*R* ^2^), selecting the lowest power for which *R* ^2^ values were close to or exceeded 0.80. Separate co-expression networks were constructed for each disease to identify distinct modules associated with disease-specific biological processes.

### 2.3 Differential gene expression and network rewiring analysis

Differential expression analysis was conducted using the *limma* package in RStudio following preprocessing and normalization of the raw expression data. Microarray data were processed using the *affy* package from Bioconductor (https://www.bioconductor.org/, accessed on 10 December 2025), applying the RMA method for normalization. Quality control of the normalized data was performed using the *arrayQualityMetrics* package, which generates an interactive HTML report including outlier detection, MA plots, boxplots, density distributions, and inter-array distance analysis. Batch effects were evaluated and corrected using the *ComBat* function from the *sva* package, and outlier detection was performed with WGCNA-based hierarchical clustering. The expression matrix was annotated with gene identifiers from the Ensembl database (http://www.ensembl.org) using the *biomaRt* package. Probes lacking valid gene annotations were excluded, and multiple probes mapping to the same gene were collapsed into a single representative expression value using the *collapseRows* function. Sample groups were defined based on phenotypic data, categorizing samples into control (CTL) and disease (DS) groups. A linear model was fitted to the normalized expression data using the *lmFit* function, and empirical Bayes moderation of the standard errors was applied via *eBayes*. Differentially expressed genes (DEGs) were identified based on an absolute log_2_ fold change (|log_2_FC|) > 1 and a false discovery rate (FDR) <0.05. Genes with positive log_2_FC values were considered significantly upregulated, while those with negative values were considered downregulated. Genes with FDR values above 0.05 were considered not significantly differentially expressed.

The top 100 genes exhibiting the highest absolute expression differences between the T2DM and HTN datasets were selected for network rewiring analysis. Pearson correlation coefficients were calculated between all pairs of selected genes within each condition to construct condition-specific co-expression networks. In these networks, nodes represent genes, and edges represent statistically significant co-expression relationships. Rewired genes were identified by quantifying each gene’s connectivity as the sum of its correlation coefficients with all other nodes in both networks. A rewiring score was defined as the difference in connectivity between conditions: Rewiring Score = Connectivity in T2DM - Connectivity in HTN. Genes with the largest absolute rewiring scores were considered to have undergone significant transcriptional reorganization. A global rewiring network was then constructed, wherein nodes represent genes and edges reflect significant changes in gene-gene correlations between T2DM and HTN.

### 2.4 Co-expression network construction and module detection

A weighted gene co-expression network analysis (WGCNA) was performed to identify gene modules associated with clinical traits in T2DM and HTN. Three expression matrices were constructed: one for T2DM (combining datasets GSE25724 and GSE20966), one for HTN (including GSE24752, GSE28345 and GSE28360), and a third integrated matrix for cross-disease analysis incorporating all five datasets. Genes were filtered by variance, retaining those above the 70th percentile, and quality control was performed using the *goodSamplesGenes* function to exclude incomplete or low-quality data. Outlier samples were identified via hierarchical clustering. Clinical traits were reviewed across all five datasets. While some included information on age and body mass index (BMI), only sex was consistently reported. As a result, sex was the only clinical variable retained and was compiled into a unified metadata file for downstream analyses. To approximate scale-free topology, the soft-thresholding power (β) was determined using the *pickSoftThreshold* function from the WGCNA package. The β value was chosen as the lowest power for which the scale-free topology model fit (*R* ^2^) was close to or exceeded 0.80. Based on this threshold, an adjacency matrix was constructed and subsequently transformed into a topological overlap matrix (TOM) for network analysis. Gene modules were identified using the *blockwiseModules* function, applying the following parameters: unsigned TOM, minimum module size of 200 genes, and a merge cut height of 0.20. Modules comprising fewer than 200 genes were excluded from further analysis. Module eigengenes (MEs) were computed and correlated with clinical traits using Pearson correlation. Modules significantly associated with clinical traits (p < 0.05) were prioritized for downstream functional and network analyses.

### 2.5 Data collection and protein-protein interaction network construction

Protein-protein interaction (PPI) networks were constructed for the most representative co-expression modules associated with T2DM and HTN using high-confidence interaction data (combined score >0.9) obtained from the STRING database (version 11.5). Two expression matrices were assembled, one for T2DM (combining datasets GSE25724 and GSE20966), and one for HTN (including GSE24752, GSE28345 and GSE28360). Selected modules consisted exclusively of protein-coding genes. Filtered interaction data was analyzed using custom Python scripts based on the NetworkX package. Key hub genes were identified according to the degree and betweenness centralities metrics. Genes ranking among the highest in connectivity were designated as network hubs. Then, community detection was performed using the greedy modularity optimization algorithm to identify major subnetworks (communities) within each PPI network. Final network visualizations were generated using Matplotlib, applying the spring layout algorithm for optimal spatial node distribution.

### 2.6 Multilayer network construction and visualization

A multilayer regulatory network was constructed to characterize the shared and disease-specific molecular architectures underlying T2DM and HTN. This network integrates five biological layers.

1. DEGs layer: DEGs shared in T2DM and HTN.
2. Co-expression modules layer: The most significant module shared in T2DM and HTN.
3. Rewired layer: Genes exhibiting significant condition-specific changes in network connectivity.
4. Transcription factor activity layer: TF activity in T2DM and HTN, respectively.

The final multilayer network was exported as a GraphML file and rendered in an interactive 3D view using Arena3Dweb (https://pavlopoulos-lab-services.org/shiny/app/arena3d) and Pyvis, enabling dynamic exploration and visualization of the cross-layer connectivity.

### 2.7 Transcription factor activity analysis

Transcription factor (TF) activity was inferred using the DoRothEA database (version 1.14.1) and the VIPER algorithm (version 1.36.0). The expression matrix was preprocessed by collapsing multiple probes per gene via mean expression values and removing duplicate gene entries. Only TF–target interactions with high-confidence scores (confidence levels A and B) were retained and converted into VIPER regulons using the dorothea_hs object. Normalized enrichment scores (NES) were computed using the *“scale”* method within the viper () function. Differential TF activity between case and control groups was assessed by two-tailed Student’s t-tests, with p-values <0.05 considered statistically significant.

### 2.8 *In silico* validation of predictive relevant genes

To explore the tissue-specific expression profiles of the most relevant genes identified in T2DM and HTN, an *in silico* validation was performed using data from the GTEx Portal (https://www.gtexportal.org/, accessed on 15 January 2025). Transcript abundance was quantified in transcripts per million (TPM), providing normalized gene expression estimates across multiple human tissues. Disease-relevant tissues were selected based on the known pathophysiology of each condition. For T2DM, the analysis included the pancreas, liver, kidney cortex, skeletal muscle, subcutaneous adipose tissue, and visceral adipose tissue. For HTN, tissues included the left ventricle, atrial appendage, coronary artery, aorta, kidney cortex, adrenal gland, and lung. Heatmaps were constructed to visualize expression gradients and to identify tissue-specific transcriptional patterns. Hierarchical clustering was applied to both genes and tissues to reveal functional groupings and highlight potential biological relevance to cardiometabolic dysfunction.

## 3 Results

### 3.1 Identification of differentially expressed genes across type 2 diabetes and hypertension

Gene expression data from three hypertension-related datasets (GSE24752, GSE28345, and GSE28360) comprising 5, 7, and 13 samples respectively, and two type 2 diabetes datasets (GSE20966 with 20 samples and GSE25724 with 13 samples) were preprocessed and batch-corrected, resulting in a unified expression matrix containing 12,251 genes across 58 samples. Differential expression analysis between T2DM and HTN samples identified eight significantly differentially expressed genes (DEGs) with an FDR <0.05 (Table 1). The upregulated genes distinguishing T2DM from HTN include *SPINK1* (Log2FC = 1.23, FDR = 1.93 × 10^−2^), which encodes a secreted serine protease inhibitor that prevents trypsin-mediated tissue damage. Next is *ANPEP* (Log2FC = 1.13, FDR = 3.98 × 10^−2^), which encodes a membrane-bound aminopeptidase involved in peptide metabolism and immune function. *MT1G* (Log2FC = 1.05, FDR = 3.26 × 10^−2^) belongs to the metallothionein family and is responsible for metal ion binding and detoxification. *NR4A1* (Log2FC = 1.02, FDR = 1.82 × 10^−2^) follows, encoding a nuclear receptor involved in cell proliferation, apoptosis, and metabolic regulation. Finally, *PRSS2* (Log2FC = 1.00, FDR = 4.07 × 10^−2^) encodes an inactive precursor of trypsin, expressed in secretory tissues such as the pancreas. Among the downregulated genes, *SCD5* (Log2FC = −1.20, FDR = 3.98 × 10^−2^) encodes a desaturase that plays a role in the biosynthesis of monounsaturated fatty acids. *QPCT* (Log2FC = −1.32, FDR = 2.21 × 10^−2^) encodes an enzyme that catalyzes the formation of pyroglutamate-modified peptides involved in neuropeptide processing. The functional annotation of these genes was obtained from the GeneCards® database (https://www.genecards.org).

**TABLE 1:** Common differentially expressed genes identified across transcriptomic profiles in T2DM and HT**N**.

Gene symbol	logFC	AveExpr	t	P. Value	FDR	B
SPINK1	1.2275	7.9677	4.0546	0.0001	0.0193	0.8893
ANPEP	1.1296	8.4200	3.2907	0.0017	0.0398	−1.2752
MT1G	1.0515	10.1510	3.4681	0.0010	0.0326	−0.7976
NR4A1	1.0155	7.2615	4.0990	0.0001	0.0182	1.0232
PRSS2	1.0006	8.0206	3.2764	0.0017	0.0407	−1.3127
SCD5	−1.2022	8.0146	−3.2899	0.0017	0.0398	−1.2771
QPCT	−1.3188	7.2413	−3.8272	0.0003	0.0221	0.2170

### 3.2 Co-expression analysis and module identification

Weighted gene co-expression network analysis was applied independently to the T2DM, HTN, and combined T2DM-HTN datasets. After filtering out low-variance genes, appropriate soft-thresholding powers were selected to achieve scale-free topology (T2DM: β = 10; HTN: β = 3; T2DM-HTN: β = 10). Network construction identified seven co-expression modules in T2DM, eight in HTN, and ten in the integrated dataset.

#### 3.2.1 Co-expression network analysis identifies a disease-linked gene module marked by metabolic and cellular transcriptional repression in T2DM and HTN

We explored convergent transcriptional programs between type 2 diabetes mellitus and hypertension by performing an integrative weighted gene co-expression network analysis (WGCNA) using transcriptomic data from GSE20966 and GSE25724 (T2DM) and GSE24752, GSE28345 and GSE28360 (HTN). This strategy enabled a unified co-expression network to capture shared molecular signatures across cardiometabolic conditions. The WGCNA revealed ten distinct modules of co-expressed genes, labeled ME0 through ME9, each summarized by its module eigengene (ME). These modules varied in size, with ME1 comprising 2,486 genes (the largest) and ME9 including 280 genes (the smallest) (Table 2). Significantly, the integrative analysis facilitated the identification of modules associated with biological processes potentially influenced by age, metabolic status, and vascular remodeling. ME7 showed the strongest association with disease status, displaying a significant negative correlation (r = −0.498, *p* = < 0.001). ME5 also exhibited a negative correlation with disease (r = −0.463, *p* = < 0.001), followed by ME4 (r = −0.264, *p* = < 0.05), though with a more modest effect. Conversely, positive correlations were observed for ME1 (r = 0.355, *p* = < 0.001), ME6 (r = 0.352, *p* = < 0.001) and ME8 (r = 0.321, *p* = < 0.01). The Modules rest did not reach statistical significance.

**TABLE 2:** Summary of co-expression modules identified by WGCNA and their correlation with clinical traits of the merged T2DM2-HTN transcriptome dataset.

Module	Number of Genes	Disease (r)	Gender (r)
ME0	2,139	0.210	0.098
ME1	2,486	0.355	−0.209
ME2	1,607	0.020	−0.162
ME3	1,495	0.065	0.039
ME4	1,447	−0.264	0.132
ME5	1,128	−0.463	0.050
ME6	690	0.352	0.002
ME7	508	−0.498	0.032
ME8	471	0.321	0.030
ME9	280	0.023	0.110

These results suggest that specific co-expression modules, particularly ME7 and ME1 capture disease-associated transcriptional programs that may be shared between T2DM and HTN. No significant associations were detected between any module and the variable *Gender*, indicating that the observed co-expression patterns are primarily driven by disease status rather than sex differences.

Functional enrichment analysis linked these modules to key biological processes such as metabolic reprogramming, vascular remodeling, and immune regulation pathways central to the pathophysiology of T2DM and HTN. Given its strong inverse association with disease status, ME7 was selected for downstream analyses. This module exhibited significant enrichment for genes involved in cellular and metabolic regulation (Supplementary Figure S1). The most significantly enriched Gene Ontology (GO) term was “cellular metabolic process,” encompassing over 259 genes and indicating broad transcriptional downregulation of essential cellular functions in the disease context. This was closely followed by the “macromolecule metabolic process” and “organic substance metabolic process,” with approximately 220 and 260 genes, respectively, highlighting widespread metabolic repression. Together, these findings suggest that ME7 captures coordinated transcriptional repression of genes essential for maintaining cellular and metabolic homeostasis in T2DM and HTN.

#### 3.2.2 Disease-specific co-expression network analysis uncovers distinct transcriptional programs in T2DM and HTN

The datasets were analyzed separately to investigate disease-specific co-expression patterns. This stratified approach allowed for the identification of distinct transcriptional modules unique to each condition, disentangling shared molecular signatures from disease-specific mechanisms.

**FIGURE 2:**
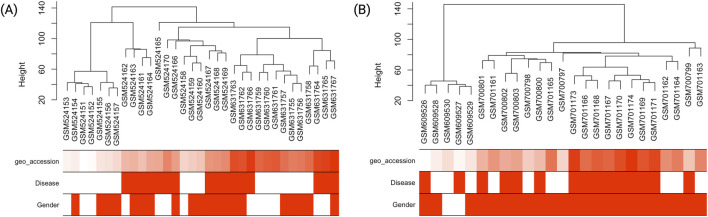
Hierarchical clustering dendrograms and associated trait heatmaps for T2DM and HTN samples. **(A)** Sample dendrogram and trait heatmap for individuals with type 2 diabetes mellitus (T2DM), displaying clustering based on gene expression profiles. Traits such as Disease (case/control) and Gender (female/male) are annotated to facilitate visual assessment of group-specific patterns. **(B)** Corresponding dendrogram and heatmap for hypertension (HTN) samples, illustrating separation between hypertensive and normotensive individuals. The hierarchical clustering reveals potential associations between transcriptomic profiles and clinical traits, serving as a foundation for downstream co-expression network analysis. Disease status: red indicates presence of disease (case), white indicates absence (control). Gender: red indicates female, white indicates male.

Hierarchical clustering of HTN samples (Figure 2A) revealed distinct segregation between hypertensive individuals and healthy controls, with the Disease trait clearly delineating the major branches of the dendrogram. This indicates that transcriptional profiles cluster according to disease status, supporting the biological relevance of the trait-based grouping and validating the quality of the expression data.

Similarly, the T2DM dataset (Figure 2B) showed a clear clustering of diabetic individuals and healthy controls. The hierarchical sample dendrogram revealed distinct groupings aligned with disease status, further supporting the robustness of the transcriptional differences. As observed in the HTN dataset, disease classification emerged as the primary driver of sample separation, while gender did not contribute significantly to the clustering pattern.

##### 3.2.2.1 ME3 in T2DM: a disease-associated co-expression module with key pathophysiological functions

The co-expression analysis of the T2DM dataset identified 13 distinct modules (MEs), each comprising different numbers of genes (Table 3). Notably, ME3 displayed a strong positive correlation with disease status (r = 0.59, p < 0.001), underscoring its potential central role in T2DM pathogenesis. Additionally, ME1 was positively correlated with disease (r = 0.52, p < 0.001). In contrast, ME6 was strongly negatively correlated with disease (r = −0.51, p < 0.001), indicating reduced expression of these genes in T2DM patients. The statistical significance of ME3 and ME1 in relation to disease reinforces their relevance to the underlying molecular mechanisms of T2DM. These findings identify ME3, ME1, and ME6 as key modules warranting further investigation as potential regulatory hubs in T2DM. Functional enrichment analysis of ME3, comprising 1,637 genes, revealed an overrepresentation of biological processes linked to metabolic activity and intracellular transport (Supplementary Figure S2). The most enriched pathways included “nitrogen compound metabolic process” (FDR = 8.99e-07) “cellular processes” (FDR = 5.65e–19), “organic substance metabolic process” (FDR = 8.53e–11), “metabolic process” (FDR = 9.61e–11), and “primary metabolic processes” (FDR = 3.62e–09).

**TABLE 3:** Summary of co-expression modules identified and their correlation with clinical Traits in the WGCNA of T2DM Dataset.

Module	Number of Genes	Disease (r)	Gender (r)
ME0	732	0.111	−0.090
ME1	4,102	0.521	−0.130
ME2	3,146	−0.004	−0.082
ME3	1887	0.595	−0.229
ME4	1,284	−0.396	0.126
ME5	1,211	0.118	0.001
ME6	780	−0.516	0.083

These results emphasize the central role of metabolic dysregulation in T2DM pathogenesis. Upregulation of genes within ME3 may initially represent a compensatory response to metabolic stress; however, sustained activation could impair insulin signaling, glucose uptake, and secretion, particularly in skeletal muscle, adipose tissue, and pancreatic β-cells. Furthermore, the enrichment in nitrogen metabolism suggests a potential link to oxidative stress and mitochondrial dysfunction. Collectively, these findings position ME3 as a critical node mediating adaptive and maladaptive responses in T2DM.

##### 3.2.2.2 ME7 as a disease-linked co-expression module underlying immune and vascular dysregulation in HTN

The co-expression analysis of the HTN dataset identified 10 distinct modules (Table 4). ME1 was the largest module (7,604 genes), whereas ME8 contained the fewest genes (375). These modules reflect distinct gene networks with coordinated expression patterns across the samples. Among these, ME7 was the most strongly associated with the “Disease” trait, displaying a robust positive correlation (r = 0.52, p = < 0.01), indicating increased expression of ME7 genes in hypertensive individuals compared to controls. Given the strong positive correlation, the biological processes enriched in ME7 are likely upregulated in HTN (Supplementary Figure S3). Functional enrichment analysis of ME7, comprising 358 genes, revealed significant processes, including “RNA processing” (FDR = 2.90e–03), “nitrogen compound metabolic process” (FDR = 2.90e–03), and “RNA metabolic process” (FDR = 2.90e–03), all showing enhanced molecular activity in hypertension.

**TABLE 4:** Summary of co-expression modules identified and their correlation with clinical traits in the WGCNA of HTN dataset.

Module	Number of Genes	Disease (r)	Gender (r)
ME0	454	−0.396	−0.026
ME1	7,604	−0.333	−0.546
ME2	3,954	0.071	0.431
ME3	3,213	0.467	0.457
ME4	749	−0.078	−0.445
ME5	687	−0.105	−0.369
ME6	553	−0.430	−0.164
ME7	417	0.520	0.326
ME8	375	−0.113	0.144

Specifically, the upregulation of “RNA metabolic process” and “nitrogen compound metabolic process” suggests a elevated cellular turnover and metabolic demand. The enrichment of these processes underscores a state of heightened transcriptional and metabolic activity, which may contribute to the vascular and systemic alterations associated with hypertension. Overall, the upregulation of ME7 in hypertensive individuals suggests activation of transcriptional programs involved in RNA processing and nitrogen metabolism, which may reflect increased biosynthetic activity and metabolic remodeling in response to hypertensive stress. These findings highlight ME7’s role in supporting the elevated molecular and metabolic demands associated with hypertension.

### 3.3 *GNB1* and *JAK1* emerge as central hubs in the PPI network, defining key functional modules in T2DM

**FIGURE 3:**
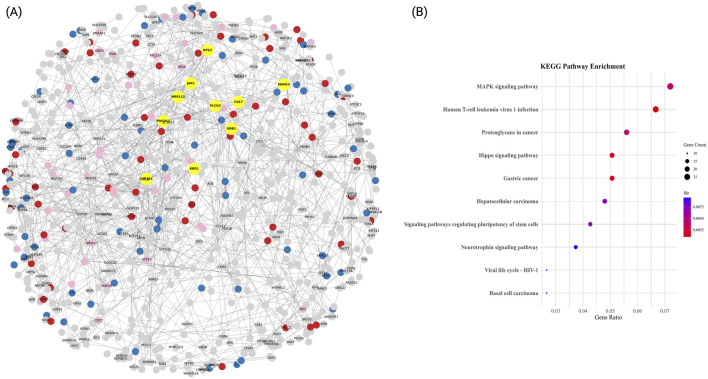
Functional PPI network and enrichment analysis of major clusters in T2DM. **(A)** High-confidence protein-protein interaction (PPI) network (confidence score >0.9) constructed using STRING. Nodes represent proteins, and edges denote experimentally validated interactions. Hub genes, identified by degree centrality, are highlighted in yellow. The three most functionally coherent communities, defined by greedy modularity clustering, are color-coded: MAPK signalling pathway (blue), Proteoglycans in cancer (red), and Hepatocellular carcinoma (pink). The remaining proteins are shown in gray. The network layout was optimized using a spring-force algorithm. **(B)** KEGG pathway enrichment for each module. Dot plots display significantly enriched terms, with dot size indicating the number of genes per term and color representing statistical significance (–log10 adjusted FDR).

The PPI network constructed for the T2DM-specific ME3 module comprised 1,637 proteins and 9,168 high-confidence interactions (confidence score >0.9), forming a highly interconnected structure with an average node degree of 5.31 (Figure 3A). Hub genes were identified based on degree centrality, with *GNB1* emerging as the principal hub, exhibiting 38 direct interactions. This finding underscores the central role of *GNB1* suggests it may serve as a central regulator of protein–protein interactions within the cellular context of type 2 diabetes mellitus.

Additional highly connected genes included *JAK1* (32 interactions), *RPS3* (32 interactions), and *MAPK3* (28), which are associated with pathways related to cytokine signaling, protein synthesis and degradation, and cell cycle regulation, respectively. Notably, the presence of other highly connected genes such as *PDCD11* (26 interactions*), KRR1* (26), *CYP2E1* (24*), MRPL13 (*24), *PLCG2* (22 interactions), and *RPS27* (22*)* further suggest the involvement of ribosome biogenesis, mitochondrial function, xenobiotic metabolism, and phosphoinositide signaling, as key contributors to T2DM pathophysiology.

#### 3.3.1 Functional enrichment of T2DM PPI communities highlights oncogenic signaling and viral response pathways

The modular organization of the T2DM-specific PPI network was explored using a modularity-based clustering algorithm, which identified 95 distinct communities. Among these, the most functionally relevant clusters were subjected to KEGG enrichment analysis (Figure 3B). The top enriched pathways included Pathways of neurodegeneration, multiple diseases (33 proteins, FDR <0.05), MAPK signaling pathway (27 proteins, FDR <0.01), and Human T-cell leukemia virus 1 infection (25 proteins, FDR <0.001). Additional enriched routes such as Proteoglycans in cancer and *Salmonella* infection (21 proteins each, both FDR <0.01) were also identified. Representative genes involved in these pathways include *GNB1, JAK1, RPS3*, *MAPK3,* and *IL6*, suggesting that transcriptional responses in T2DM converge on inflammatory, oncogenic, and neurodegenerative signaling axes.

### 3.4 Hypertension PPI network reveals ribosomal and ubiquitin-mediated regulation as key pathways

**FIGURE 4:**
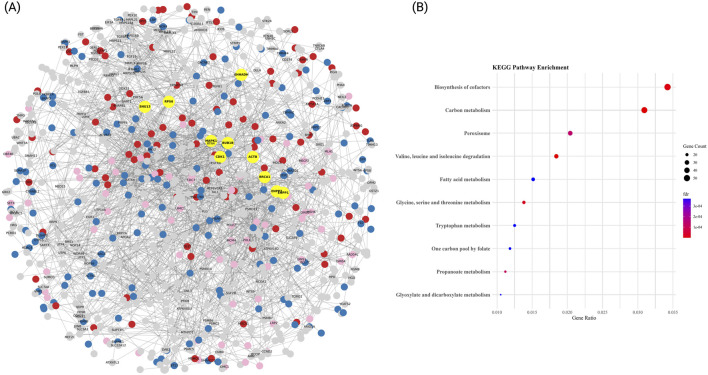
PPI network and functional enrichment analysis in HTN. **(A)** High-confidence protein-protein interaction (PPI) network (confidence score >0.9) based on STRING database data. Nodes represent proteins, and edges indicate experimentally validated interactions. Hub genes, identified by degree centrality, are highlighted in yellow. The top three functional communities, detected via greedy modularity clustering, are color-coded: Enriched in energy and simple-substrate metabolism pathways (blue), Enriched in central cofactor and amino-acid metabolic pathways (red), and Enriched in peroxisomal and short-chain fatty-acid pathways (pink). The remaining proteins are shown in gray. The network layout was optimized using a spring-force algorithm to enhance interpretability. **(B)** KEGG pathway enrichment for each module. Dot plots display significantly enriched Gene Ontology biological processes, KEGG pathways, and molecular functions for each cluster. Results highlight key mechanisms in HTN pathogenesis, including protein degradation, immune signaling, and translational regulation. Dot size represents the number of genes per term, while color denotes statistical significance (–log10 adjusted FDR).

We further investigated the biological relevance of the ME7 and ME3 modules associated with HTN by constructing a high-confidence PPI network (Figure 4A). To build this network, we merged the ME7 (417 genes) with the ME3 module (3,213 genes) and selected the top 1,000 genes for analysis. The resulting network consisted of 1,000 nodes and 4,109 edges, with an average node degree of 20.51 and 2 connected components. Hub genes identified within ME7 and ME3 included *JUN* (degree = 172), *BRCA1* (degree = 132), *MAPK1* (degree = 124), *FOS* (degree = 120), *CREBBP* (degree = 112), *NOP56* (degree = 94), *NDC80* (degree = 78), *RAD51* (degree = 74), and *DCTN1* (degree = 74). To further characterize the functional structure of the network, community detection was performed, identifying three principal clusters based on KEGG pathway enrichment analyses (Figure 4B): Amino acid and carbon metabolism cluster: This cluster exhibited strong enrichment in metabolic pathways such as valine, leucine and isoleucine degradation, carbon metabolism, and fatty acid metabolism, with gene counts between 23 and 47 and –log_10_(FDR) values exceeding 4.5.

Peroxisomal and cofactor biosynthesis cluster: This group showed enrichment in pathways including peroxisome, biosynthesis of cofactors, and one carbon pool by folate, indicating active redox and coenzyme regulation mechanisms (gene counts between 18 and 52). Glycine, tryptophan and propanoate processing cluster: This cluster was enriched for glycine, serine and threonine metabolism, tryptophan metabolism, and propanoate metabolism, with modest gene ratios but consistent statistical support (adjusted p-values <0.001).

Together, these communities illustrate distinct but interconnected molecular programs disrupted in HTN. The combined dysregulation of amino acid metabolism, carbon and lipid processing, and cofactor biosynthesis provides insights into the complex transcriptional architecture underlying disease progression in hypertensive patients.

### 3.5 Network rewiring and multi-scale analysis reveal perturbations in molecular interactions in T2DM and HTN

**FIGURE 5:**
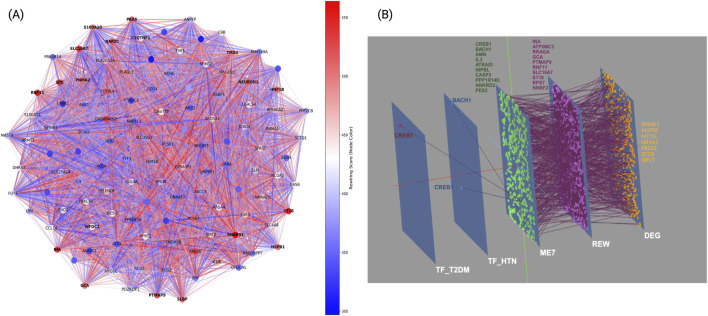
Network rewiring and multi-scale analysis in T2DM and HTN. **(A)** Node color corresponds to the rewiring score, where positive values (red) indicate network expansion and increased connectivity, while negative values (blue) reflect network contraction and loss of interactions. **(B)** Three-dimensional multilayer network in T2DM and HTN. Panel B, depicts five colored planes, each corresponding to a distinct network layer: the dark-blue plane corresponds to transcription factors in T2DM, the light-blue plane to transcription factors in HTN, the green plane to shared co-expression module, the purple plane to genes with the strongest rewiring scores, and the orange plane to shared DEG. Nodes on each plane share its color, and inter-layer edges connect identical genes across planes, allowing you to follow any given gene through its roles in transcription factor networks, co-expression modules, rewiring analysis and differential expression.

Network rewiring analysis was performed to investigate structural remodeling of molecular interactions in T2DM and HTN (Figure 5A). This approach quantified topological changes within condition-specific co-expression networks. Each gene was assigned a rewiring score based on the difference in connectivity and correlation strength between the two conditions. Positive scores reflected increased connectivity in T2DM relative to HTN, whereas negative scores indicated reduced connectivity in T2DM or disrupted co-expression relationships. The analysis revealed a heterogeneous rewiring pattern, with a subset of hub genes displaying significant gains or losses of interactions. In T2DM, genes such as *ST18, SNAP91*, and *RAP2C* exhibited increased connectivity (rewiring score >500; 95th percentile), suggesting a role in adaptive responses to metabolic stress and vesicle trafficking. Additionally, *SLBP, RNF11*, and *NEUROD1* showed increased connectivity, potentially reflecting alterations in RNA binding, ubiquitin signaling, and neuronal regulation. Conversely, genes such as *SLC16A7, SPX*, and *PAX8* showed reduced connectivity (rewiring score <215; 5th percentile), implicating disrupted monocarboxylate transport, neuropeptide signaling, and transcriptional regulation in T2DM pathophysiology. Meanwhile, in HTN, rewiring events predominantly affected inflammatory and translational pathways. Notably, hub genes such as *S100A10, HSPB1*, and *WFDC2* exhibited reduced connectivity (rewiring score < −15), suggesting disruptions in immune regulation, stress response, and extracellular protease inhibition. Additionally, genes such as *C1QTNF1* and *C1R*, involved in the complement cascade, also showed connectivity losses, pointing toward altered inflammatory signaling.

These findings indicate that HTN pathophysiology involves network remodeling affecting immune and translational regulation, potentially contributing to vascular dysfunction through both transcriptional and post-translational mechanisms.

To further integrate transcriptional, topological, and expression-level alterations, we constructed a multilayer network connecting transcription factors differentially active in T2DM or HTN, shared co-expression modules, genes with high rewiring scores, and differentially expressed genes (DEGs). The most significant module identified by co-expression analysis was ME7, enriched in genes involved in immune-metabolic regulation and mitochondrial function (Figure 5B).

At the transcriptional level, *CREB1* and *BACH1* displayed distinct and context-specific connectivity. *CREB1*, a transcription factor implicated in glucose metabolism and immune regulation, was exclusively active in the T2DM layer and connected to multiple genes within shared co-expression module and the rewiring layer, suggesting a pivotal regulatory role in T2DM-driven network remodeling. Conversely, *BACH1*, a known modulator of oxidative stress and vascular tone, was prominent in the HTN-specific layer, interfacing with the same module but through distinct targets, indicating disease-specific regulatory programs.

The shared co-expression module, enriched in immunometabolic genes, served as a convergence point for both conditions, connecting upstream transcriptional regulators with rewired genes. Within the rewiring layer, topological hubs such as *ST18, RNF11, SLC16A7*, and *PTMAP9* integrated transcriptional signals with downstream expression alterations, indicating their roles as dynamic mediators of structural network shifts across both diseases.

Finally, in the DEG layer, metabolic and stress-response regulators including *SPINK1*, *ANPEP*, *MT1G*, *NR4A1*, *PRSS2*, *SCD5*, and *QPCT* emerged as shared downstream effectors. These genes are involved in proteolysis, lipid metabolism, and oxidative stress, suggesting that both T2DM and HTN converge on pathways related to cellular stress adaptation and metabolic regulation, despite being initiated by distinct upstream mechanisms.

### 3.6 Transcription factor activity reveals shared and distinct regulators in T2DM and HTN

**FIGURE 6:**
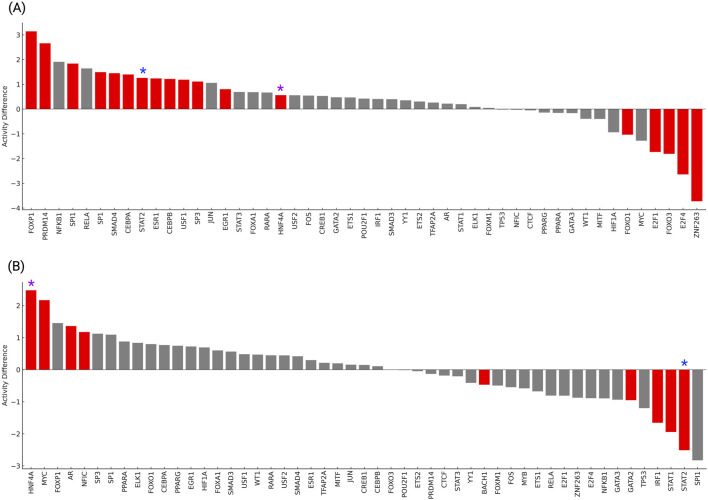
Transcription factor activity differences in T2DM **(A)** and HTN **(B)** were inferred using VIPER and high-confidence DoRothEA regulons. Red bars indicate TFs with significantly altered activity (p < 0.05). Activity differences are represented as normalized enrichment scores (NES), where positive values denote increased activity and negative values denote repression in disease compared to controls.

The analysis of master transcription factor (TF) activity profiles revealed distinct yet partially overlapping regulatory signatures in T2DM and HTN (Figure 6). In T2DM (Figure 6A), significant activation of metabolic and epigenetic regulators was observed, including *PRDM14*, *FOXP1*, *CEBPA*, and *SP1* (*p < 0.01*). These factors are involved in transcriptional control of metabolic, developmental, and immune processes, suggesting their coordinated upregulation in diabetic tissue. Conversely, *E2F4, FOXO3, E2F1*, and *ZNF263* all show significantly decreased activity (*p* < 0.01). These transcription factors are associated with cell cycle progression, DNA repair, and transcriptional regulation. Their inhibition in T2DM may reflect a downregulation of proliferative and repair pathways, potentially contributing to the pathophysiology of metabolic dysfunction and insulin resistance.

Our results show that in HTN (Figure 6B), transcription factor activity analysis revealed significant activation of key regulators including *MYC, AR, HNF4A*, and *NFIC* (*p* < 0.05). These transcription factors are associated with metabolic regulation, hormone signaling, and vascular homeostasis, suggesting their potential roles in the pathophysiology of hypertension. Conversely, strong repression was observed for *IRF1, STAT1, STAT2, and GATA2*, all displaying significantly decreased activity in hypertensive individuals compared to controls (*p* < 0.05). These factors are typically involved in immune surveillance and inflammatory responses, and their suppression may reflect impaired immunoregulatory mechanisms contributing to vascular dysfunction. Together, these findings highlight the presence of coordinated transcriptional activity changes in HTN, shared regulators identified across both conditions included *HNF4A, MYC, FOXO1* and *STAT2*, all exhibiting significant dysregulation (p < 0.05). Specifically, *HNF4A* was significantly activated in both T2DM and HTN, suggesting its consistent involvement in metabolic regulation and transcriptional reprogramming associated with cardiometabolic stress. *FOXO1* displayed opposite activity patterns, being repressed in T2DM and activated in HTN, suggesting divergent responses to oxidative and metabolic stress in each condition. *MYC* showed repression in T2DM but activation in HTN, highlighting context-dependent regulation of proliferative and metabolic control pathways.

*STAT2* was activated in T2DM but strongly repressed in HTN, suggesting opposing role in immune modulation and inflammatory signaling. These findings reveal the presence of shared yet differentially regulated transcriptional networks between T2DM and HTN. The identification of HNF4A as a commonly activated factor supports its potential as a therapeutic target in the overlapping molecular landscape of metabolomic and hypertension disorders.

### 3.7 Tissue-specific expression patterns of hub genes in T2DM and HTN: implications for metabolic and cardiovascular dysfunction

**FIGURE 7:**
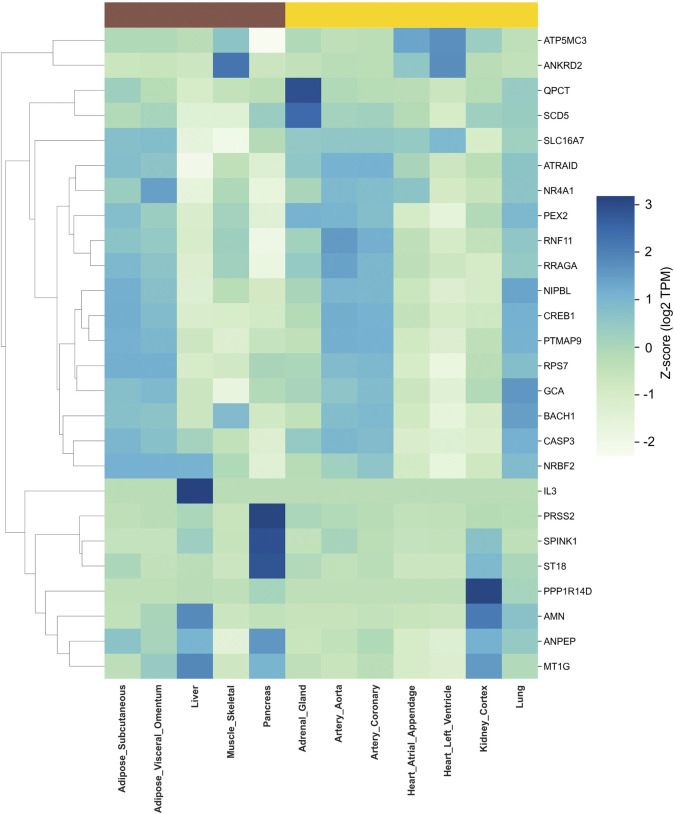
Tissue-specific expression of hub genes in T2DM and HTN. The heat maps display the transcripts per million (TPM) values of key hub genes across human tissues based on GTEx RNA-seq data. Hierarchical clustering was performed to identify tissue-specific expression patterns. The left panel represents hub genes associated with T2DM, showing predominant expression in metabolic tissues such as the pancreas, liver, and adipose depots. The right panel illustrates hub genes implicated in HTN, with enriched expression in vascular and cardiac tissues, including the aorta, coronary arteries, and heart compartments.

Leveraging RNA-seq data from the GTEx database, we assessed the tissue-specific expression patterns of hub genes identified in T2DM and HTN, revealing distinct transcriptional signatures that align with the metabolic and vascular pathophysiology of each condition. Hierarchical clustering and heatmap visualization facilitated the identification of organ-specific molecular programs (Figure 7; Supplementary Table S1).

Analysis of GTEx baseline expression profiles in T2DM-related tissues showed that *PRSS2* (Z = 3.12) and *SPINK1* (Z = 2.89) attain their highest levels in pancreas, whereas *QPCT* (Z = 2.92) and *SCD5* (Z = 2.43) are most strongly expressed in adrenal gland. Skeletal muscle exhibits peak expression of *ANKRD2* (Z = 2.25), and adipose depots are characterized by elevated *RPS7* in subcutaneous fat (Z = 1.17) and *NR4A1* in visceral omentum (Z = 1.44).

Among tissues linked to hypertension, *PPP1R14D* (Z = 3.12) and *AMN* (Z = 2.12) show maximal expression in kidney cortex, while *PTMAP9* (Z = 1.16), *RRAGA* (Z = 1.37) and *CREB1* (Z = 1.21) are most abundant in the aorta. Coronary artery demonstrates moderate enrichment of *ATRAID* (Z = 1.11), and *ANPEP* exhibits a primary peak in pancreas (Z = 1.58) alongside secondary vascular expression. Finally, *MT1G* reaches its highest Z-score in liver (Z = 1.86), consistent with its role in systemic homeostasis.

Because GTEx data reflect healthy “baseline” expression, these shifts upregulation of pancreas and kidney enriched genes (*SPINK1, PRSS2, ANPEP*) and downregulation of adrenal or metabolic tissue–enriched genes (*QPCT, SCD5*), highlight how T2DM and HTN jointly perturb organ-specific pathways (exocrine stress, oxidative balance, lipid processing and vascular remodeling).

## 4 Discussion

Recent systems biology studies have employed integrative omics approaches to explore the molecular complexity of type 2 diabetes mellitus, hypertension, and comorbidities (Kamali et al., 2022; Lei et al., 2023; Rönn et al., 2024). This study developed a comprehensive computational framework that integrates transcriptomic data, co-expression module analysis, protein-protein interaction networks, network rewiring, and transcription factor activity inference to uncover shared and disease-specific molecular signatures.

In this study, age was the only clinical variable consistently available across all transcriptomic datasets, justifying its inclusion in the analysis. However, inconsistent reporting of key clinical factors, such as BMI, HbA1c, blood pressure, and sex limited their integration and introduced potential confounding variables affecting transcriptomic patterns in T2DM and HTN. BMI significantly influences the expression of inflammatory and metabolic genes in adipose and vascular tissues (Wensveen et al., 2015; Fang et al., 2015), while HbA1c and blood pressure impact endothelial and immune-related transcriptional responses (Bayaraa et al., 2022; Chen et al., 2021). Although sex was included in some datasets, its inconsistent reporting led to its exclusion from analysis, despite its known effects on gene expression and disease risk (Shi et al., 2024). To enhance biological interpretability and the translational impact of systems-level analyses in cardiometabolic research, future studies should prioritize standardized clinical metadata. Among the shared DEGs in T2DM and HTN, several genes were significantly upregulated, including *SPINK1, ANPEP, MT1G, NR4A1*, and *PRSS2*. These genes are involved in protease inhibition, aminopeptidase activity, oxidative stress response, and inflammatory signaling, suggesting activation of stress-adaptive and immune pathways in both conditions. In particular, *NR4A1*, a nuclear receptor sensitive to metabolic and inflammatory cues, may represent a key transcriptional node linking metabolic stress with vascular dysfunction (Martínez-González et al., 2021).

Conversely, *SCD5* and *QPCT* were significantly downregulated. *SCD5*, a lipogenic enzyme, plays a role in fatty acid desaturation, and its reduced expression points to altered lipid homeostasis (Igal and Sinner, 2021). *QPCT*, implicated in peptide processing and neuroendocrine signaling, may contribute to impaired vascular tone or hormonal regulation when suppressed (Zhu et al., 2022).

Together, these expression patterns suggest a transcriptional convergence between T2DM and HTN, characterized by chronic inflammation, oxidative stress, and metabolic disruption, potentially contributing to their frequent comorbidity and shared vascular complications.

**FIGURE 8:**
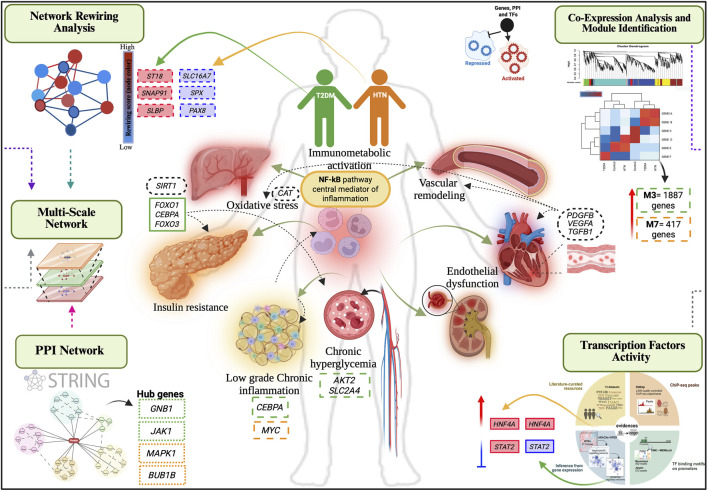
Integrative network model of shared mechanisms in T2DM and HTN. Multi-scale integrative network analysis reveals shared and disease-specific mechanisms connecting type 2 diabetes mellitus (green) and hypertension (orange). Rewired genes, indicated with dashed-line rectangles, show increased connectivity in red and decreased connectivity in blue. Hub genes are framed with densely dashed rectangles, while transcription factors are highlighted with solid-line rectangles, with upregulated factors in red and repressed factors in blue. Co-expression module genes are framed with long dashed rectangles and differentially expressed genes are framed with black, dotted line rectangles.

Our findings reveal a robust transcriptional convergence in T2DM and HTN centered on vascular remodeling, immune activation, and metabolic stress responses. This multi-layered approach identified key co-expression modules associated with T2DM (ME3) and HTN (ME7), each enriched for genes involved in endothelial dysfunction, chronic inflammation, oxidative stress, and remodeling of the extracellular matrix. Hub genes such as *GNB1*, *JAK1*, *JUN*, and *BRCA1*, as well as key transcriptional regulators including *HNF4A*, and *STAT2,* emerged as central nodes within disrupted regulatory circuits (Figure 8).

Unlike previous transcriptomic studies that focused primarily on differentially expressed genes, our approach integrated dynamic regulatory features through network rewiring analysis and TF activity profiling (Chiang et al., 2018; Ali et al., 2022; Farrim et al., 2024). This strategy supports the notion that cardiometabolic diseases share core regulatory networks and highlights novel molecular targets for precision therapeutic strategies.

Our analysis revealed convergent inflammatory and vascular remodeling pathways in both diseases. The T2DM-associated module (ME3) was enriched in proinflammatory signaling, particularly *TNF* and *NF-κB* pathways, alongside oxidative stress and intracellular transport. These findings are consistent with studies reporting that low-grade chronic inflammation within pancreatic islets contributes to T2DM by impairing β-cell function and insulin secretion (Dludla et al., 2023). In contrast, the HTN-associated module (ME7) was primarily enriched in genes involved in mitochondrial function, carbon metabolism, and fatty acid oxidation, suggesting that metabolic stress and redox imbalance are key transcriptional features in hypertensive pathology (Krzemińska et al., 2022). While vascular remodeling and immune activation are widely recognized contributors to hypertension, our data highlights a prominent role for energy metabolism in ME7. These findings complement previous work showing that hypertension-associated loci influence vascular and immune cell programs (Skovgaard et al., 2025). Notably, *KMT2A* has been identified as an upstream epigenetic regulator of hypertension onset, pointing to chromatin remodeling as a potential mediator of vascular dysfunction.

Our results suggest that prolonged metabolic stress and hemodynamic load may converge on common molecular pathways, particularly NF-*κ*B signaling and oxidative stress responses, promoting *β*-cell failure and vascular injury. The oxidative stress-related genes found in ME3 support this model, as hyperglycemia and lipotoxicity are known to increase reactive oxygen species (ROS) production, which activates NF-*κ*B and upregulates inflammatory cytokines (Keane et al., 2015; Li et al., 2016; Vilas-Boas et al., 2021; Mukai et al., 2022).

This ROS-driven inflammatory cascade has been implicated in *β*-cell dedifferentiation and dysfunction in transcriptomic and experimental models (Trojanowski et al., 2020; Khin et al., 2021). Similarly, vascular oxidative stress contributes to endothelial dysfunction in hypertension through pathways involving *EGFR* and proinflammatory kinases (Rodrigues-Diez et al., 2015; Higashi, 2022). While our analysis did not identify *EGFR* as a central hub, the most significant co-expression module in HTN showed enrichment in oxidative and mitochondrial pathways. Together, these findings support the hypothesis that inflammation and remodeling pathways act as mechanistic bridges linking T2DM and HTN, corroborating epidemiological evidence of their frequent co-occurrence (Motuma et al., 2023).

The PPI network analysis identified key hub genes associated with T2DM and HTN, revealing regulatory signatures that highlight disease-specific molecular alterations. In the T2DM-specific network (ME3), *GNB1* emerged as the most connected hub, consistent with its established role in transducing G protein-coupled receptor (GPCR) signals that regulate insulin secretion, glucose metabolism, and inflammatory pathways (Saddala et al., 2018; Murakami et al., 2019). Its centrality reinforces recent transcriptomic and functional data implicating *GNB1* in pancreatic stress responses and insulin resistance, two hallmarks of T2DM pathogenesis. Additional hubs in the T2DM network included *JAK1*, *RPS3*, *MAPK3*, these hubs underscore the convergence of inflammatory signaling, translational control, and metabolic dysregulation in the pathogenesis of T2DM. *JAK1* mediates cytokine-driven inflammation and contributes to insulin resistance in adipose tissue (Huang et al., 2024), while *RPS3* links translational control with NF-κB activation, promoting oxidative stress in β-cells. This suggests that in type 2 diabetes, proinflammatory and oxidative stress mechanisms are activated, affecting key tissues involved in the disease’s pathophysiology.

In the HTN-specific network (ME7), *MAPK1* was identified as the topological hub with the highest degree of connectivity, followed by *BUB1B*, *CDK1*, and *RPS6*. The prominence of *MAPK1,* a key effector of the MAPK/ERK pathway, highlights its relevance in hypertensive pathophysiology, where it modulates vascular smooth muscle cell proliferation, endothelial function, and oxidative stress responses. Previous studies have shown that *MAPK1* activation contributes to vascular remodeling and increased vascular tone, and its dysregulation has been implicated in blood pressure elevation (Potthoff et al., 2016; Liu and Lin, 2022).

*BUB1B* and *CDK1,* key regulators of mitosis and cell cycle progression, likely reflect proliferative vascular responses and endothelial turnover in hypertension (Bolanos-Garcia and Blundell, 2011). Meanwhile, *RPS6*, a translational regulator and mTOR target, suggests increased protein synthesis and stress adaptation. Together, these hubs point to coordinated cell cycle and biosynthetic activation as key features of vascular remodeling in hypertensive states.

Transcriptional rewiring involves changes in gene interaction patterns within co-expression or regulatory networks, irrespective of their expression levels. A high rewiring score indicates significant shifts in a gene’s connectivity, which may suggest changes in its regulatory or functional role due to disease-related stimuli. This analysis can reveal important changes that traditional methods might miss (Zipeto et al., 2015; Shepherd et al., 2023; Yan and Huangfu, 2022). Both increased and decreased connectivity can be biologically significant. Genes losing centrality may be silenced or disconnected from key regulatory programs, while those gaining interactions might be integrating into stress response or disease-specific pathways.

In type 2 diabetes, genes like *ST18*, *SNAP91*, and *SLBP* are more central in the network, showing their importance in dealing with ongoing metabolic stress. *ST18* is a transcription factor linked to inflammation and may make pancreatic β-cells more vulnerable under high blood sugar (Henry et al., 2014). *SNAP91* may affect how insulin is recycled (Naudi-Fabra et al., 2024), while *SLBP* helps stabilize histone mRNA, supporting cell growth and changes in the cell’s structure. These changes suggest that the body tries to adjust metabolic and cellular processes to protect β-cell function. The involvement of exocrine-associated genes in endocrine dysfunction supports the hypothesis of crosstalk between pancreatic compartments, a concept gaining recognition in diabetes research (Hu et al., 2025).

In contrast, hypertension shows that genes like *SLC16A7*, *SPX*, and *PAX8* are less central, indicating they play a reduced role in the vascular and kidney networks. *SLC16A7* reduced role may impact how energy is used in blood vessels (Kirat and Kato, 2015). *SPX* decreased connectivity suggests problems in communication between nerves and blood vessels (Kumar et al., 2021). *PAX8* reflects changes in controlling sodium and fluid levels, which are important for managing blood pressure (Zivotic et al., 2022). These trends highlight a loss of coordination among metabolic, vascular, and kidney systems in hypertension. This supports growing evidence that vascular dysfunction in HTN arises from the interplay between impaired energy metabolism, peptide signaling, and renal-endothelial crosstalk (Cicalese et al., 2021).

Importantly, these rewiring patterns were not evident in the differential expression analysis alone, highlighting the added value of topology-based methods in capturing dynamic regulatory events. Genes that maintain stable expression levels can still undergo significant changes in their network positioning, acquiring or losing interactions that alter their functional context. This network-centric perspective provides a deeper understanding of disease mechanisms beyond static transcript abundance (Vignoli et al., 2020).

The construction of a multilayer network integrating differentially expressed genes, rewired genes, and transcription factor activity revealed a modular architecture that connects T2DM and HTN through shared and distinct regulatory hubs (De Domenico, 2023; Chen and Padi, 2024). Hub genes such as *GNB1*, *JAK1*, and *RPS3* dominated the T2DM network layer, suggesting critical roles in inflammatory signaling, insulin resistance, and translational control under metabolic stress. In contrast, *MAPK1*, *BUB1B*, and *CDK1* were central hubs in HTN, implicating them in vascular signaling, cell cycle regulation, and stress-responsive remodeling. Moreover, integrating regulatory activity scores from master transcriptional analysis (VIPER) revealed key TFs, including *CREB1* and *BACH1*, as potential upstream regulators orchestrating the observed network remodeling. These TFs may mediate both diseases’ convergent transcriptional responses to inflammation, oxidative stress, and metabolic imbalance. This multilayer network model advances the current understanding of the molecular interplay between T2DM and HTN by distinguishing static interaction patterns from dynamic connectivity shifts.

TF activity analysis revealed both disease-specific and shared regulatory programs. In T2DM, *FOXP1*, *PRDM14, SPI1, SP1, SMAD4, STAT2, ESR1, CEBPB, USF1, SP3, EGR1* and *HNF4A* were significantly activated, in line with their roles in inflammatory signaling, metabolic regulation, and transcriptional reprogramming in insulin-responsive tissues (Bočkor et al., 2024; Matsui et al., 2024). Conversely, *ZNF263, E2F4, FOXO3, E2F1, *and *FOXO1* were repressed, suggesting key pathways controlling cell cycle arrest, oxidative stress response, and apoptosis may be suppressed in insulin-resistant tissues, potentially reflecting a compensatory adaptation to chronic metabolic overload or a shift toward survival mechanisms under cellular stress. In HTN, a distinct TF activity profile emerged, with prominent activation of *HNF4A, MYC, AR*, and *NFIC* factors implicated in vascular remodeling, endothelial proliferation, and transcriptional reprogramming of metabolic and inflammatory genes (Gao et al., 2017; Norambuena-Soto et al., 2020; Wan et al., 2022). *STAT2, STAT1, IRF1, GATA2* and *BACH1* were also repressed in HTN, reinforcing their possible involvement in immune regulation, interferon signaling, and antioxidant defense mechanisms that may be suppressed during chronic vascular stress. Two TFs, *HNF4A*, and *STAT2,* exhibited significant and contrasting activity profiles across T2DM and HTN. *HNF4A* was strongly activated in T2DM and moderately activated in HTN, consistent with its role in hepatic glucose metabolism and vascular gene regulation (Thymiakou et al., 2023; Chai et al., 2019). In contrast, *STAT2* was activated in T2DM but markedly repressed in HTN, suggesting a divergent interferon signaling response (Gothe et al., 2022). These opposing patterns suggest that while both diseases share inflammatory components, the nature and direction of immune signaling may differ, with T2DM engaging antiviral and metabolic immune pathways, and HTN exhibiting immune suppression that is potentially linked to chronic vascular stress or immune exhaustion.

These findings collectively delineate a transcriptional regulatory landscape shaped by disease-specific and overlapping master regulators. Identifying *HNF4A* and *STAT2* as standard hubs across T2DM and HTN underscores their potential as dual-disease targets in metabolic and vascular pathologies. This convergence is consistent with integrative analyses of GWAS loci and transcriptomic networks linking inflammatory signaling and metabolic regulation (Fernández-Tajes et al., 2019).

The expression analysis of hub genes, the co-expression module most relevant to them, rewired genes, and transcription factors across normal tissues provides critical functional context for their roles in T2DM and HTN. Hub genes from the T2DM network, such as *RNF11* and *GCA*, exhibited elevated expression in adipose depots and pancreas (Z-scores >0.5), supporting their involvement in insulin signaling, vesicle trafficking, and β-cell responses to metabolic stress (Azmi and Seth, 2005). In contrast, HTN-associated genes, such as *RPS7* and *QPCT*, showed higher expression in arterial tissues (aorta and coronary arteries) and lungs, consistent with their functions in translational regulation and vascular remodeling. For instance, *RPS7* displayed Z-scores >0.9 in coronary arteries and >1.1 in subcutaneous adipose tissue, while *QPCT* was upregulated in adrenal gland tissue, a key endocrine regulator of blood pressure (Villa et al., 2024).

Furthermore, TFs including *HNF4A* and *STAT2* showed broad expression across metabolic (liver and pancreas) and cardiovascular (aorta and left ventricle) tissues. This reinforces their proposed role as upstream regulators orchestrating shared inflammatory and metabolic programs across both diseases.

These findings underscore the relevance of tissue context in interpreting molecular signatures and highlight the value of integrating transcriptomic data with expression atlases to identify therapeutic targets across cardiometabolic diseases.

However, it is important to acknowledge methodological limitations related to the transcriptomic platform employed in this study. Microarrays are widely used in transcriptomic analyses, but they have significant limitations compared to modern platforms like RNA-Seq (Lowe et al., 2017). Their lower sensitivity and restricted dynamic range hinder accurate detection of transcripts with very low or high expression levels due to signal saturation and hybridization noise. Additionally, because they rely on pre-designed probes for known transcripts, microarrays cannot identify novel genes or unannotated transcripts, limiting their role in biomarker discovery and exploration of new regulatory pathways. Nevertheless, they remain valuable in functional genomics due to the availability of historical datasets, cost-effectiveness, and reliable performance in comparative studies (Dopazo, 2006).

This study shows connections between gene expressions and conditions like type 2 diabetes and hypertension. However, these connections are only correlative. They do not prove that changes in gene expression cause these diseases. Instead, the changes are likely responses to diseases. To truly understand the role of certain genes in T2DM and HTN, we need more experiments, such as RT-qPCR or gene knockdown. Until we conduct these studies, we cannot confirm that the hub genes or transcription factors we identified directly cause the onset or progression of these diseases. Therefore, future research is necessary to understand the importance of these genes and their networks in heart and metabolic health.

## 5 Conclusion

Our multiscale network analysis reveals shared transcriptional and regulatory mechanisms between type 2 diabetes mellitus and hypertension, centered on inflammation, oxidative stress, and metabolic dysregulation. Key hub genes and transcription factors, including *HNF4A* and *STAT2*, emerged as potential standard drivers, suggesting the existence of central nodes mediating the comorbidity between these diseases. These findings open new avenues for developing shared biomarkers and targeted therapeutic strategies for convergent molecular processes. Further experimental validation will be essential to confirm their clinical relevance and translational potential.

## Data Availability

The datasets presented in this study can be found in online repositories. The names of the repository/repositories and accession number(s) can be found in the article/Supplementary Material.
