# Supplementary figures and images for: Shared transcriptional regulators and network rewiring identify therapeutic targets linking type 2 diabetes mellitus and hypertension
Source: Front Mol Biosci. 2025 Aug 20;12:1621413. doi: 10.3389/fmolb.2025.1621413 (PMC12404935; doi:10.3389/fmolb.2025.1621413)

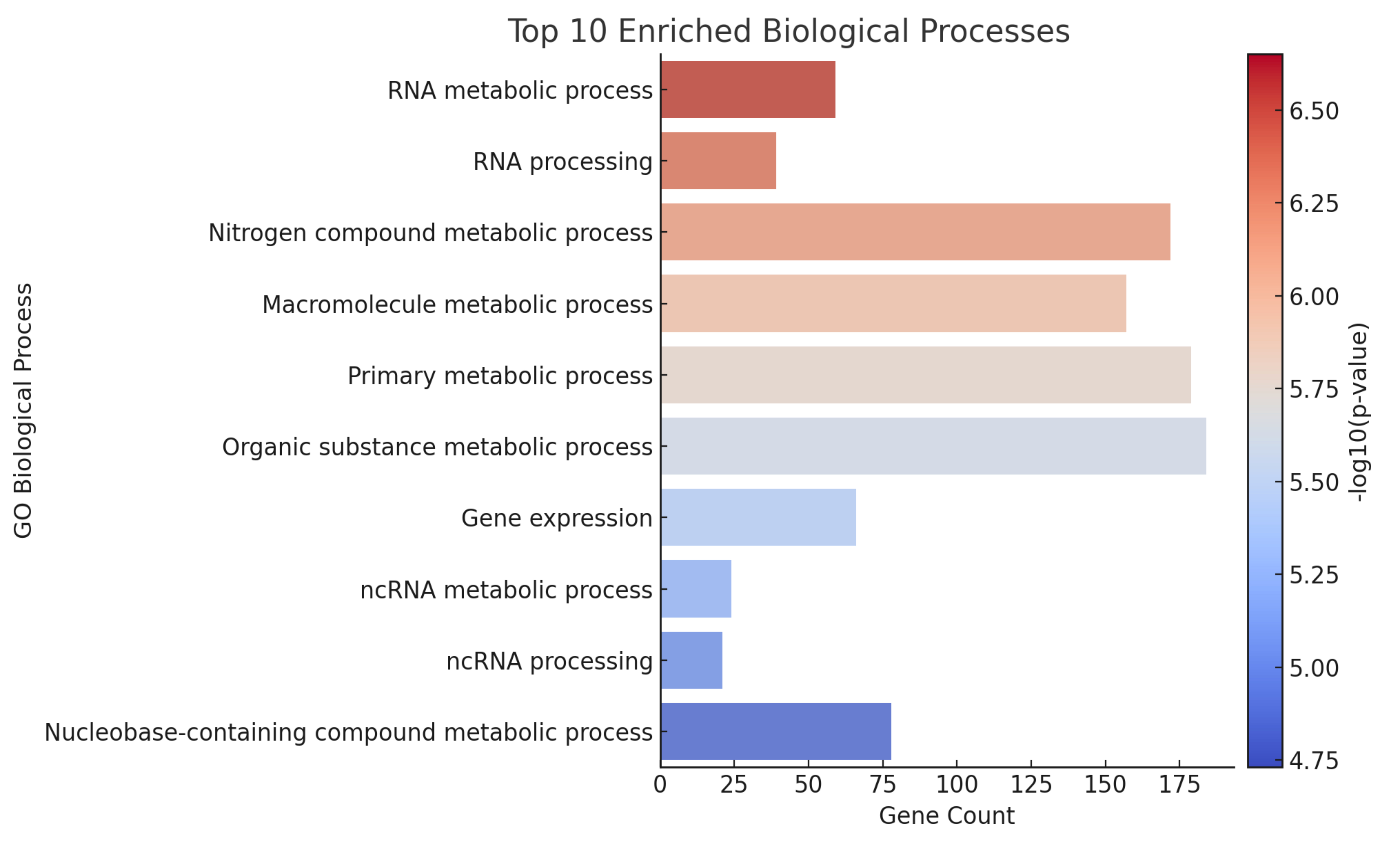

Supplement: Supplementary file 1 [file Image3.jpeg]

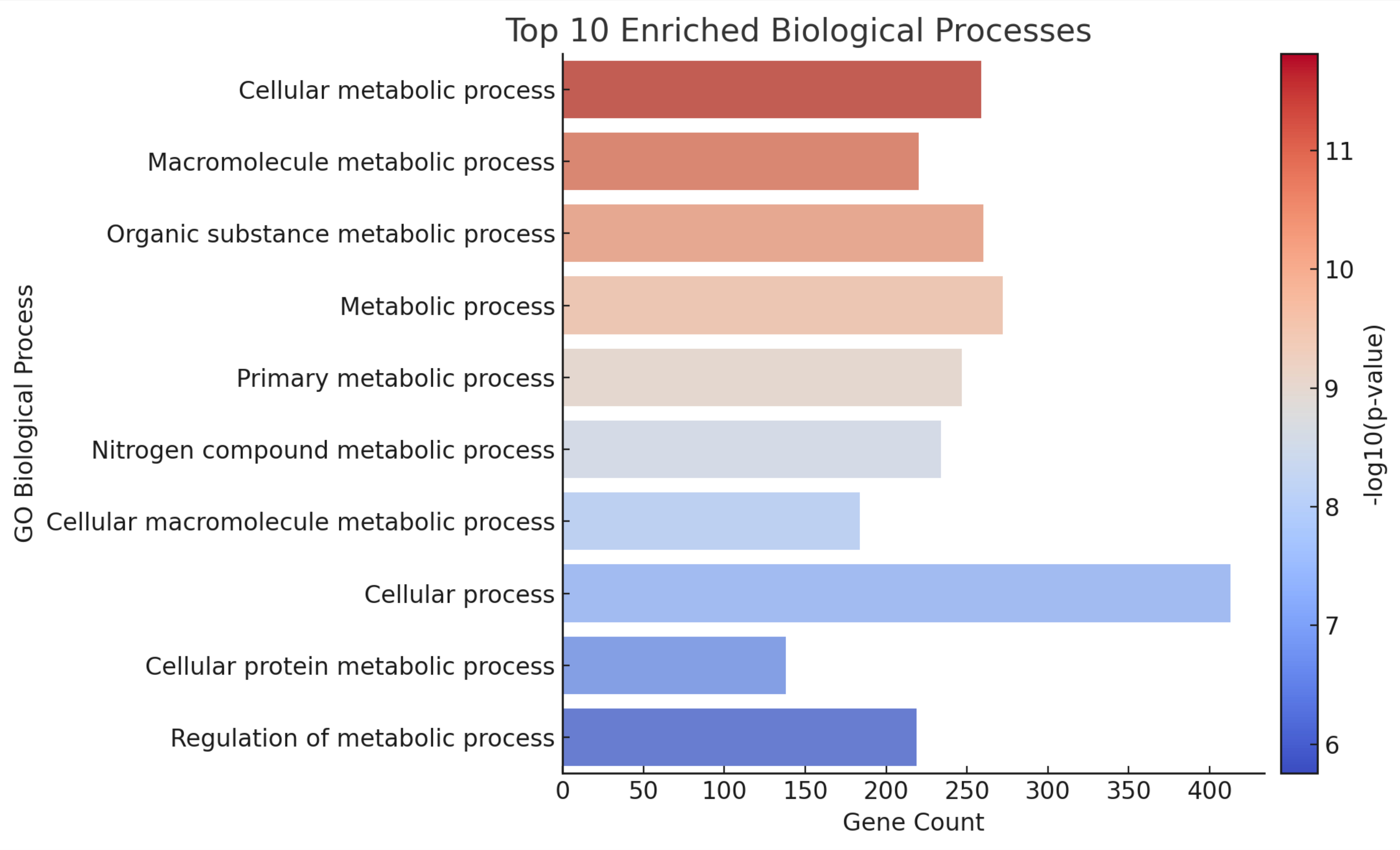

Supplement: Supplementary file 2 [file Image1.jpeg]

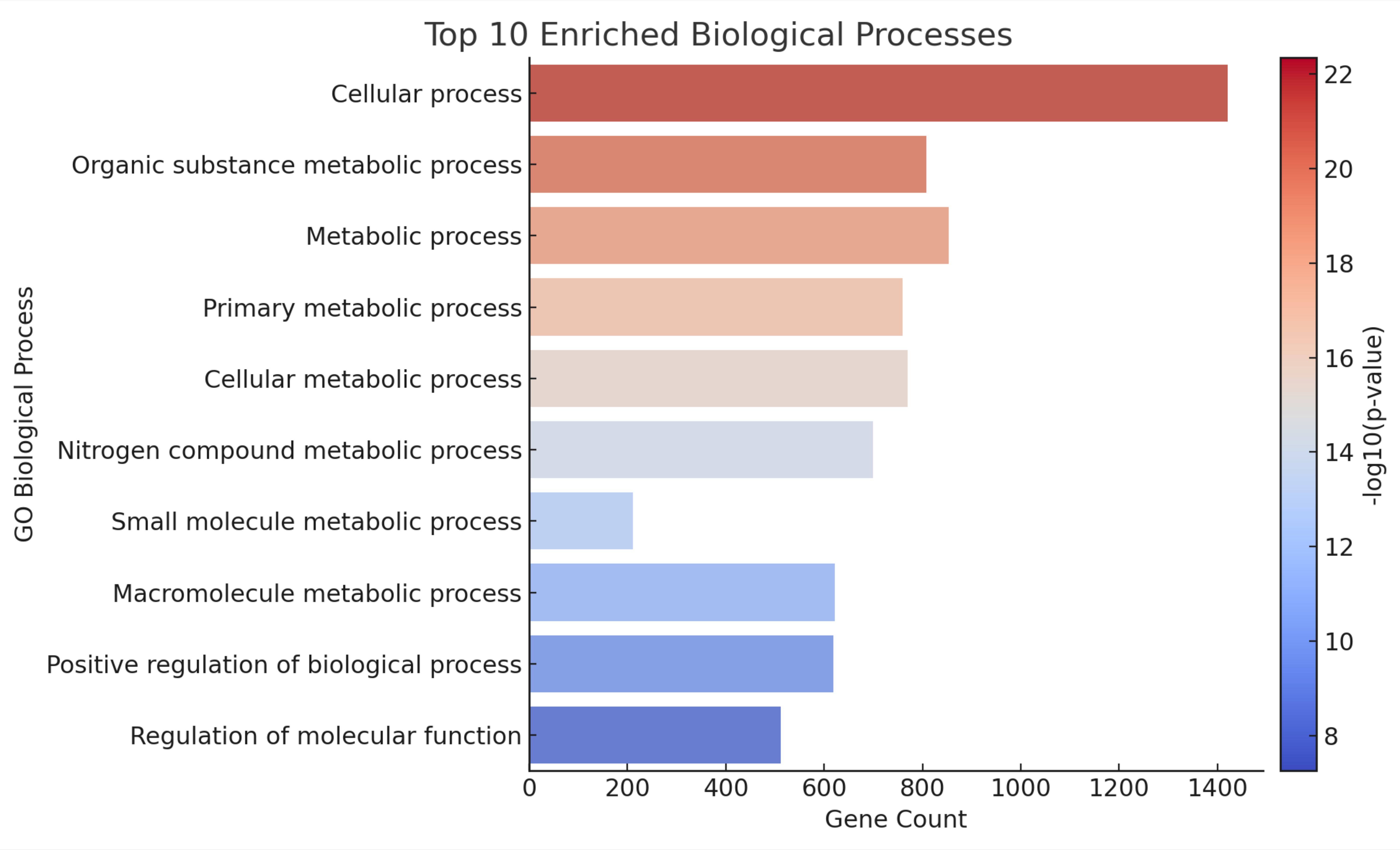

Supplement: Supplementary file 3 [file Image2.jpeg]
